# Gegen Qinlian decoction prevents post-ERCP pancreatitis by regulating NLRP3 inflammasome-mediated pyroptosis

**DOI:** 10.3389/fphar.2025.1588585

**Published:** 2025-06-20

**Authors:** Yurong Cui, Jinxin Li, Mengqiang Cai, Yu Zhang, Bing Zhao, Junying Liu

**Affiliations:** ^1^ Gastroenterology Department, The First Affiliated Hospital of Henan University of CM, Zhengzhou, Henan, China; ^2^ First Clinical Medical College, Henan University of Chinese Medicine, Zhengzhou, Henan, China; ^3^ Pathology Department, The First Affiliated Hospital of Henan University of CM, Zhengzhou, Henan, China

**Keywords:** pancrestitis, post ERCP pancreatitis, Chinese medicine (CM), Gegen Qinlian decoction (GQD), NLRP3

## Abstract

**Objective:**

Post-ERCP pancreatitis (PEP) is a type of acute pancreatitis (AP) and the most common complication after ERCP. NLRP3 inflammasome-dependent pyroptosis is a key factor in the pathogenesis of acute pancreatitis. Gegen Qinglian decoction (GQD) is a traditional Chinese medicine formula that has the effects of clearing heat and detoxifying, and is now widely used in pancreatic diseases. We have clinically found that GQD can effectively prevent PEP. To explore its potential preventive mechanism, we conducted rat experiments.

**Methods:**

Before modeling, GQD, indomethacin suppository, NLRP3 inhibitor, etc. were used for intervention, and the GQD used was qualitatively analyzed by UPLC-QTOF-MS/MS. Then, a PEP model was established by injecting contrast medium into the pancreatic and bile ducts of rats, an *in vitro* model was made by intervening with sodium taurocholate (STC) in AR42J cells. The severity of pancreatitis and the levels of NLRP3 inflammasome components were subsequently evaluated *in vivo* and *in vitro*.

**Results:**

UPLC analysis of GQD identified 227 known metabolites. The expression of NLRP3, Caspase-1, and GSDMD in PEP model was significantly increased compared to the blank group, indicating the activation of NLRP3 inflammasome mediated cell pyroptosis during PEP occurrence. Prophylactic administration of GQD resulted in a reduction in the expression of NLRP3, Caspase-1, and GSDMD. Additionally, GQD significantly alleviated the pathological inflammation in the pancreas, and reduced levels of amylase and inflammatory cytokines. The GQD group, indomethacin suppository group, NLRP3 inhibitor group, and siRNA gene suppression group showed similar results.

**Conclusion:**

GQD can effectively prevent PEP, and its primary mechanism may be related to the regulation of NLRP3 inflammasome-mediated pyroptosis. Provide theoretical basis for the use of traditional Chinese medicine in clinical practice to prevent diseases.

## 1 Introduction

Endoscopic retrograde cholangiopancreatography (ERCP), as a pivotal interventional therapy in gastrointestinal endoscopy, plays a crucial role in the diagnosis and treatment of various biliary and pancreatic disorders (Pekgöz, 2019). However, as an invasive therapeutic technique, its complications such as post-ERCP pancreatitis (PEP), biliary tract infection, bleeding, and perforation are increasingly significant in clinical practice. Post-ERCP pancreatitis (PEP) is a form of acute pancreatitis (AP), and it represents the most common and serious complication following ERCP (ASGE Standards of Practice Committee et al., 2017). The incidence of PEP is approximately 9.7%, but in high-risk populations such as young women, sphincter of Oddi dysfunction, history of prior pancreatitis, and difficult cannulation of the papilla of Vater, the incidence can be as high as 15% (Cahyadi et al., 2022). Up to 5% of cases experience severe PEP and may develop life-threatening complications, including multiple organ failure, peripancreatic fluid accumulation, and up to 1% of cases result in death (Cahyadi et al., 2022).

At present, the pathogenesis of PEP is not fully understood, mainly due to various factors ultimately leading to pancreatic enzyme activation, which in turn causes damage to pancreatic acinar cells (PACs) and inflammatory reactions. Therefore, the inflammatory response and oxidative stress caused by excessive release of inflammatory factors play a key pathological role in the occurrence and development of PEP. Among them, NLRP3 inflammasome dependent pyroptosis is a key factor in the pathogenesis of acute pancreatitis, the necrosis related molecule GSDMD may be an independent prognostic biomarker for AP (Al Mamun et al., 2022). Multiple studies have shown consistent pyroptosis induced pancreatic acinar cell death during acute pancreatitis. When damaged by pyroptosis, acinar cells rupture and release cellular contents and inflammatory factors, exacerbating the inflammatory response (Wang et al., 2020; Gao et al., 2021).

In clinical practice, prevention is the primary focus for PEP, oral or rectal administration before ERCP can effectively reduce the occurrence of PEP. At present, non steroidal anti-inflammatory drugs (NSAIDs) are commonly administered through the rectum to prevent PEP in clinical practice. However, there are differences in clinical efficacy, and there are many contraindications and side effects such as gastrointestinal ulcers, gastrointestinal bleeding, and liver, liver, and kidney dysfunction (Cahyadi et al., 2022). Therefore, finding more effective drugs to prevent PEP is currently one of the hotspots in ERCP research.

Traditional Chinese medicine (TCM) has accumulated theoretical and empirical knowledge over thousands of years in the realm of “preventing disease before it occurs”. Gegen Qinlian decoction (GQD) originates from the classic formula in *Treatise on Febrile Diseases* (*Shang Han Lun* in Chinese), composed of dry root of *Pueraria montana* var. *lobata* [Fabaceae; Pueraria montana radix et rhizoma] (Gegen), dry root of *Scutellaria baicalensis* Georgi [Lamiaceae; Scutellaria baicalensis radix et rhizoma] (Huangqin), dry rhizomes of *Coptis chinensis* Franch. [Ranunculaceae; Coptis chinensis radix et rhizoma] (Huanglian), and dry root of *Glycyrrhiza uralensis* Fisch. [Fabaceae; Glycyrrhiza uralensis radix et rhizoma] (Gancao). The plant name were checked in http://mpns.kew.org/mpns-portal/. GQD has long been used to treat abdominal pain and fever, with the functions of clearing heat and detoxifying, and is now widely used in pancreatic diseases. Studies have shown that GQD can regulate exocrine pancreatic function, thereby affecting the prognosis of chronic pancreatitis (He and Yang, 2022). Research has shown that in diabetic rats, intervention with Gegen Qinlian decoction (GQD) significantly reduces inflammatory cytokines, indicating that GQD can systematically improve pancreatic inflammatory responses (Xu et al., 2020; Ren et al., 2021). We used GQD to prevent PEP based on the basic principles of traditional Chinese medicine, and have achieved good clinical efficacy. In this study, we aim to investigate GQD as an innovative therapeutic agent for preventing PEP, and explore relevant molecular mechanisms based on the role of NLRP3 inflammasome in AP.

## 2 Materials and methods

### 2.1 Materials and reagents

Pre-administration of rectal NSAID indomethacin before ERCP is a recommended prophylactic measure for PEP according to the European Society of Gastrointestinal Endoscopy (ESGE) guidelines (Dumonceau et al., 2020). The indomethacin suppositories used in this study were purchased from Hubei Dongxin Pharmaceutical Co., Ltd. (Hubei, China). MCC950 inhibits both canonical and non-canonical activation of the NLRP3 inflammasome without affecting K^+^ efflux in macrophages, and it is a commonly used small-molecule inhibitor of the NLRP3 inflammasome (Shao et al., 2015). The MCC950 used in this study was purchased from GLPBIO (California, United States). The components of Gegen Qinlian Decoction (GQD) including Puerariae Lobatae Radix (batch number A3020012), Scutellariae Radix (batch number A3090822), and Coptidis Rhizoma (batch number A1090182) were obtained from Guangdong Yifang Pharmaceutical Co., Ltd. (Foshan, China). Glycyrrhizae Radix et Rhizoma (batch number 21029191) was purchased from Beijing Kangrentang Pharmaceutical Co., Ltd. (Beijing, China). Botanical drugs are certified and stored by the Department of pharmacy, the First Affiliated Hospital of Henan University of traditional Chinese medicine.

The amylase test kit, IL-1β, IL-18, and IL-10 ELISA kits were purchased from Elabscience Biotechnology Co.,Ltd. (Wuhan, China). The PCR kit was obtained from TOYOBO Co., Led. (Osaka, Japan). Cleaved-caspase1 Polyclonal Antibody was provided by ImmunoWay Biotechnology Co.,Ltd. (Texas, United States). Caspase-1 Antibody is provided by Affinity Biosciences Co.,Ltd. (Jiangsu, China). The NLRP3 Polyclonal Antibody and β-ACTION Antibody were provided by GeneTex Inc. (Texas, United States).

### 2.2 Preparation and chemical characterization of GQD

Gegen Qinlian Decoction (GQD) was prepared according to the adult prescription dosage of 39 g of raw botanical drugs: 15 g of Gegen, 9 g of Huangqin, 9 g of Huanglian, and 6 g of Gancao. The formula particles were dissolved in 50% methanol water at the required volume and diluted to concentrations of 20, 50, 100, 200, and 200 times, respectively. The main chemical metabolites in the GQD granules were qualitatively characterized using ultra-performance liquid chromatography coupled with quadrupole time-of-flight mass spectrometry (UPLC-QTOF-MS/MS) (Zhou et al., 2017).

### 2.3 Quality control of GQD

The quality control of GQD was carried out by HPLC. A YMC triart C18 HPLC column (4.6 mm × 250 mm, 5 μ m) was used for chromatographic separation. The mobile phase consisted of 3.5 mmol/L sodium heptanesulfonate (containing 0.1% formic acid) (solvent a) and acetonitrile (containing 0.1% formic acid) (solvent B).

0.5 g GQD was dispersed by adding appropriate amount of methanol, sonicated for 15 min, fixed volume of methanol to 100 mL, and filtered. The concentrations of liquiritin, glycyrrhizic acid, palmatine hydrochloride, daidzein, wogonin, berberine hydrochloride, daidzein, wogonin, coptisine hydrochloride, and baicalin prepared with methanol were 0.05 mg/mL, 0.05 mg/mL, 0.025 mg/mL, 0.025 mg/mL, 0.025 mg/mL, 0.1 mg/mL, 0.005 mg/mL, 0.005 mg/mL, 0.025 mg/mL, 0.15 mg/mL. The content of main components in GQD was calculated by using the relevant standard curve.

### 2.4 Animals

Seven-week-old adult male Sprague-Dawley (SD) and Specific Pathogen Free (SPF) rats, weighing 230–270 g, were purchased from the Experimental Animal Service Center of Henan University of Chinese Medicine. The animals were housed under standard conditions at room temperature (20°C–24°C) with free access to food and water. All procedures in this study were conducted in accordance with the guidelines for the care and use of laboratory animals published by the National Institutes of Health (NIH Publication No. 85-23, revised 2011). The experimental protocols were approved by the Animal Ethics Committee of Henan University of Chinese Medicine (Approval No. IACUC-202307030).

### 2.5 Drug treatment and establishment of PEP model in rats

The rats were randomly divided into six groups, with 10 rats in each group: (a) control group, (b) PEP model group, (c) PEP model + indomethacin suppository group, (d) PEP model + GQD group, (e) PEP model + MCC950 group, and (f) PEP model + MCC950 + GQD group. Starting 3 days before the procedure, the GQD group was orally administered GQD at a dosage of 1 mL/100 g body weight (0.132 g/mL), while the other groups received physiological saline at 1 mL/100 g body weight, administered once daily via gavage. The indomethacin suppository group (15 mg/kg) received rectal administration immediately after anesthesia before the procedure. The MCC950 groups received an intraperitoneal injection of MCC950 at 10 mg/kg body weight starting 3 days before the procedure.

The PEP model was established as follows: Rats were fasted for 24 h prior to the procedure but allowed free access to water. All groups, except for the normal control group, were anesthetized with an intramuscular injection of 3% pentobarbital sodium. Under sterile conditions, a midline incision was made in the upper abdomen to expose the duodenum by tracing along the stomach. The bile-pancreatic duct, identifiable by its white appearance at the mesenteric attachment of the duodenum, was located. The bile-pancreatic duct near the hepatic hilum was clamped with a vascular clamp, and a G-tube needle was directly inserted into the duct near its opening in the duodenum. A constant pressure of 50 mmHg (1 mmHg = 0.133 kPa) was applied via a sphygmomanometer to inject a contrast agent (3% iohexol, approximately 0.4 mL, OMNIPAQUE, China) into the bile-pancreatic duct over a duration of 2 min. After the injection, the clamp on the hepatic hilum was released, and the duct near the duodenum was clamped for 10 min before being released. Finally, the abdominal cavity was closed with double-layer sutures (Sherman et al., 1994).

### 2.6 Sampling

Twenty-four hours after the grouping and model establishment, the rats were anesthetized, and the abdominal cavity was opened to expose the abdominal aorta. Blood was collected from the abdominal aorta and centrifuged at 4°C and 3,000 rpm for 15 min. The upper serum layer was collected and aliquoted into sterile EP tubes, then stored at −80°C. After blood collection, the rats were euthanized, and their pancreatic tissues were harvested and fixed in 10% formalin solution.

### 2.7 Measurement of amylase and inflammatory factors

Each serum sample of 50 μL was taken, along with portions of pancreatic tissue. Amylase levels were measured using a fully automated biochemical analyzer and expressed in units per liter (U/L). The detection of various inflammatory factors, including interleukin-1β (IL-1β), IL-10, and IL-18, in both serum and pancreatic tissue was performed according to the instructions provided in the double-antibody sandwich enzyme-linked immunosorbent assay (ELISA) kits.

### 2.8 Real-time quantitative polymerase chain reaction (RT-qPCR) was used to detect the mRNA expression of NLRP3, Caspase-1, and GSDMD in pancreatic tissue

Trizol reagent (Thermo, China) was used to extract total RNA from pancreatic tissue for assessment of RNA integrity and concentration. The RNA was reverse transcribed into cDNA using a first-strand cDNA synthesis kit. β-actin served as the internal reference gene. Primers were designed by Primer Designer and synthesized by Suzhou Jinweizhi Biotechnology Co., Ltd. (primer sequences are listed in Table 1). The cDNA was amplified using a PCR machine under conditions specified in the kit’s instructions. Each sample was run in triplicate, and the average Ct value was calculated from three independent experiments. The results were analyzed using the relative quantification method (2^−ΔΔCT^ method).

**TABLE 1:** Primer sequences.

Gene	Primer sequences	Gene ID	Length (bp)
NLRP3-F	CTC​ACC​TCA​CAC​TCC​TGC​TG	287362	122
NLRP3-R	AGA​ACC​TCA​CAG​AGC​GTC​AC		
caspase-1-F	GAC​CGA​GTG​GTT​CCC​TCA​AG	25166	108
caspase-1-R	GAC​GTG​TAC​GAG​TGG​GTG​TT		
GSDMD-F	CAG​AAC​CAG​TGT​CTG​GCA​GT	315084	86
GSDMD-R	ACG​TTG​CAT​GAT​CTC​CCA​GG		
NLRP3-F	CTC​ACC​TCA​CAC​TCC​TGC​TG	287362	122
NLRP3-R	AGA​ACC​TCA​CAG​AGC​GTC​AC		
ACTB-F	ATG​GAT​GAC​GAT​ATC​GCT​GC	81822	150
ACTB-R	CTT​CTG​ACC​CAT​ACC​CAC​CA		

F: forward, R: reverse; Primer sequences species: rat.

### 2.9 Western blot was conducted to detect the protein expression of NLRP3, Caspase-1, and GSDMD in pancreatic tissue

Weighed lyophilized pancreatic tissue was pulverized using liquid nitrogen and a mortar to extract total protein. Protein concentration was determined, and samples were prepared to a concentration of 4 g/L in 1× Phosphate Buffered Saline (PBS). An appropriate amount of protein was loaded for electrophoresis. Electrophoretic transfer to PVDF membranes was performed at a constant voltage of 110 V for 90–120 min. The membranes were blocked with 5% non-fat milk in 1× TBST (Tris-buffered saline with Tween-20) for 2 h at room temperature. Primary antibodies were added and incubated overnight at 4°C. After washing the membrane three times with 1× TBST, secondary antibodies were applied and incubated at room temperature for 1 h. The membrane was washed again three times with 1× TBST, followed by detection using ECL (Enhanced Chemiluminescence) reagent in a dark room. Image Pro Plus software was used for data analysis of the saved original images, measuring grayscale values and calculating the relative grayscale values of the target protein normalized to the internal reference protein.

### 2.10 Histological analysis

After blood collection, pancreatic head tissues were collected and fixed in 10% neutral buffered formalin, while pancreatic tail tissues were stored in liquid nitrogen for further use. Three samples were selected from each group. The pancreatic head tissues underwent routine paraffin embedding, resulting in 6-μm sections. These sections were stained with hematoxylin and eosin (HE), dehydrated, cleared, coverslipped, and examined under a light microscope. Finally, all slides were observed using an optical microscope (Olympus BX53). Referring to the Schmidt evaluation criteria (Schmidt et al., 1992), histopathological scoring of pancreatic tissue inflammation was performed based on four main criteria: edema, acinar necrosis, hemorrhage, and inflammatory infiltration.

### 2.11 Immunohistochemical detection

Tissue slices were attached to anti slip glass slides and baked at 68°C for 90 min. Sequentially dewaxing and dehydrating with xylene and ethanol gradient. Incubate with 3% H_2_O_2_ solution at room temperature for 15 min and wash with PBS three times. In citrate buffer, antigen repair was performed at high pressure 95°C for 5 min, followed by washing twice with PBS. Goat serum was incubated at room temperature for 10 min to block the serum. Incubate the primary antibody at 4°C overnight and wash with PBS three times. Incubate the secondary antibody at room temperature for 30 min and wash with PBS three times. Under a microscope, color develop 100 μ L DAB solution for 5–10 min, and rinse with tap water to terminate the reaction. Stain the cell nucleus with hematoxylin for 2 min, dehydrate with ethanol gradient, clear with xylene, and seal with neutral gum. Observe and image the stained tissue sections using a microscope (Olympus).

### 2.12 Cell culture and treatment

Rat pancreatic acinar cells (AR42J) were purchased from starfish Biology (Suzhou, China) and cultured in medium supplemented with 10% fetal bovine serum. AR42J cells were treated with sodium taurocholate (STC) for 30 min, and the release rate of LDH was detected using lactate dehydrogenase (LDH) colorimetric test box (Wuhan yilairuite Biotechnology Co., Ltd., Wuhan, China), and the appropriate concentration was selected to construct the acute pancreatitis model. The appropriate concentration of GQD was selected through CCK8 experiment and then the corresponding medium was added. NLRP3 siRNA was designed by sidirect v2.0 and synthesized by universal biosynthesis, and the silencing efficiency was verified by qPCR. The most efficient siRNA was used to silence NLRP3 gene expression. After the detection of amylase, lipase, NLRP3 related qPCR and Western blot, the trypsin activity was detected by fluorescence substrate method.

### 2.13 Statistical analysis

The quantitative data are presented as mean ± standard deviation. One-way analysis of variance (ANOVA) was used for comparisons among multiple groups, followed by Turkey’ test for comparisons between two groups. Statistical analyses were performed using GraphPad Prism 10.1.2. Differences were considered statistically significant when p < 0.05.

## 3 Results

### 3.1 UPLC analysis of GQD

**FIGURE 1:**
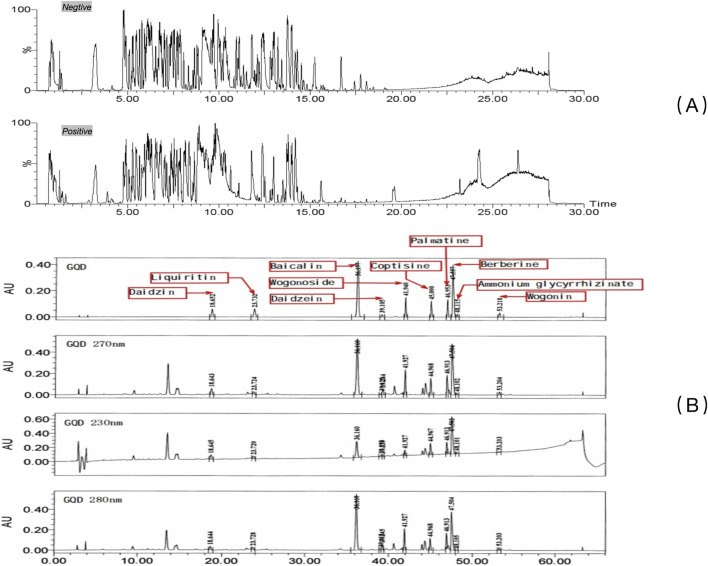
UPLC of GQD. **(A)** Qualitative characterization of major chemical metabolites in GQD; **(B)** Quality control of GQD

The overall results after UPLC-QTOF-MS/MS analysis are shown in Figure 1. Analysis and identification of the detected metabolites revealed a total of 227 known metabolites, as detailed in Supplementary Table S1. Based on previous studies on the active ingredients of GQD (Lu et al., 2022), 10 main metabolites were identified, including ammonium glycyrrhizinate, baicalin, berberine, coptisine, daidzein, daidzin, liquiritin, palmatine, wogonin, wogonoside, etc. Detailed results for these ten metabolites are provided in Table 2, and the mass spectra with ion fragments are shown in Supplementary Figure S1. UPLC quality control analysis of GQD showed that the concentrations of the above 10 metabolites were 0.6669%, 3.959%, 2.2881%, 0.5809%, 0.0763%, 0.4484%, 0.2577%, 0.5747%, 0.0843%, 0.07803%, respectively.

**TABLE 2:** Analysis results of 10 metabolites.

Metabolite	Ionic mode	Response	Observation retention time (min)	Observational m/z	Measured molecular mass (Da)	Molecular mass (Da)	Mass number error (mDa)	Theoretical fragment ions found	Adduct
Baicalin	pos	407448	9.73	447.0936	446.0863	446.0849	1.4	34	+H
	neg	383363	9.73	445.0787	446.086	446.08491	1	36	−H
Ammonium glycyrrhizinate	pos	26711	13.01	840.4385	839.4312	839.4303	0.9	63	+H
Berberine	pos	997374	10.38	336.1242	336.1247	336.1236	1.1	15	−e
Coptisine	pos	2067914	9.82	320.0931	320.0936	320.0923	1.3	10	−e
Daidzin	pos	545995	6.83	417.1187	416.1114	416.1107	0.7	50	+H, +Na
	neg	671408	6.84	461.1094	416.1112	416.11073	0.4	29	+HCOO, −H, +Cl
Daidzein	pos	637143	6.81	255.065	254.0577	254.0579	−0.2	15	+H
	neg	372752	6.81	253.0509	254.0582	254.05791	0.3	10	−H
Liquiritin	pos	23238	9.29	419.1333	418.1261	418.1264	−0.3	4	+H
	neg	85278	9.29	417.1202	418.1275	418.12638	1.1	4	−H
Palmatine	pos	982080	9.81	353.1592	352.1519	352.1549	−3	21	+H
Wogonin	pos	971670	14.19	285.0755	284.0682	284.0685	−0.3	15	+H
	neg	482487	14.19	283.0614	284.0687	284.06847	0.2	7	−H
wogonoside	neg	1213274	10.38	459.0934	460.1007	460.10056	0.1	35	−H
	pos	788044	10.34	461.109	460.1017	460.1006	1.1	31	+H, +Na

Pos: positive; neg: negative.

### 3.2 Effects of GQD on amylase and inflammatory cytokines in rats injected with iohexol into the bile-pancreatic duct

**FIGURE 2:**
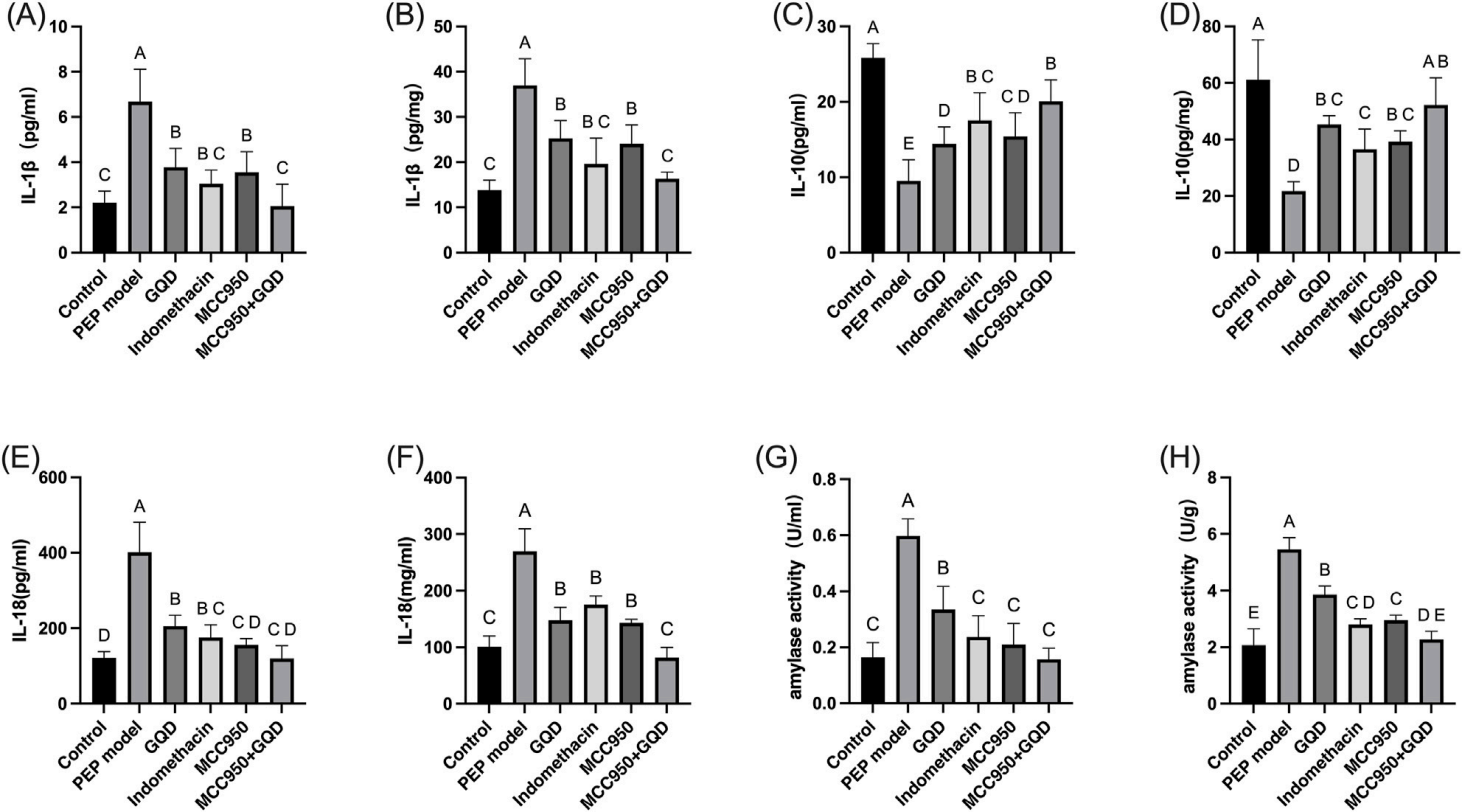
Expression of serum inflammatory factors and amylase in each group. **(A)** Serum amylase; **(B)** Tissue amylase; **(C)** Serum IL-1β; **(D)** Tissue IL-1β; **(E)** Serum IL-10; **(F)** Tissue IL-10; **(G)** Serum IL-18; **(H)** Tissue IL-18.

After sample collection, the levels of amylase in serum and pancreatic tissue were measured. In the PEP model group, both amylase and inflammatory cytokines were significantly elevated compared to the control group. In the GQD-treated group, levels of the inflammatory cytokines IL-1β and IL-18 were significantly reduced compared to the PEP model group (p < 0.05), while IL-10 levels were significantly increased (p < 0.05). There were no significant differences in inflammatory cytokines between the GQD-treated group and the PEP + Indomethacin group (p > 0.05), as shown in Figure 2.

### 3.3 The pathological effects of GQD on pancreatic tissue in rats injected with iohexol into the pancreatic bile duct

**FIGURE 3:**
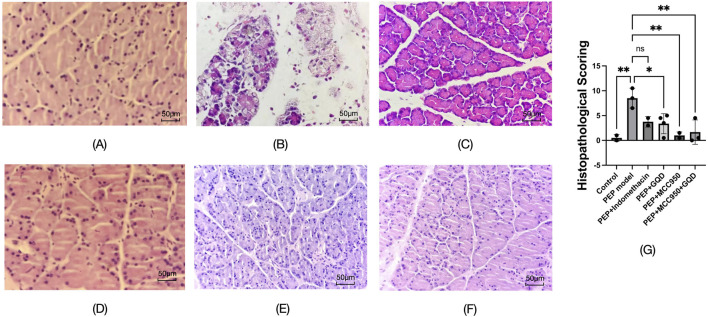
Pathological manifestations of each group (HE; ×400). **(A)** Control; **(B)** PEP model; **(C)** PEP+Indomethacin; **(D)** PEP+GQD; **(E)** PEP+MCC950; **(F)** PEP+MCC950+GQD; **(G)** Histopathological score.

To induce the PEP model, 3% iohexol was injected into the bile-pancreatic duct of the rats. Histopathological examination of the tissue sections revealed interstitial edema in the pancreatic tissue, accompanied by moderate infiltration of neutrophils and a small number of lymphocytes, along with focal acinar cell degeneration, indicative of acute inflammation (Figure 3B). Neutrophil infiltration was observed in all groups except the control group, as detailed in Figure 3. Histopathological scoring was performed on inflammation of pancreatic tissue, and the other groups were significantly lower than the model group (Figure 3G).

### 3.4 The effect of GQD on NLRP3-Caspase1 cell pyroptosis in rats injected with iohexol into the pancreatic bile duct

**FIGURE 4:**
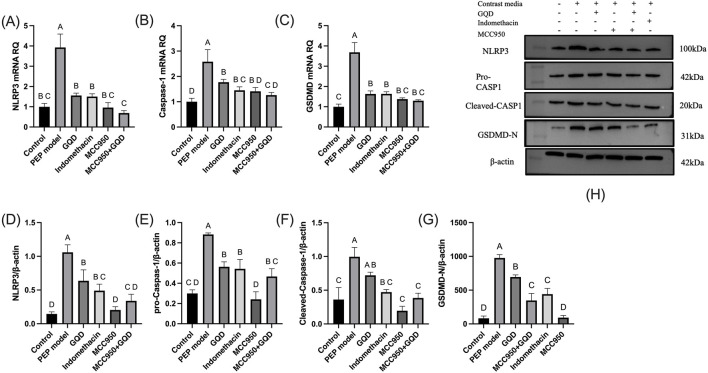
NLRP3 inflammasome expression in each group. **(A)** NLRP3 mRNA; **(B)** Caspase-1 mRNA; **(C)** GSDMD mRNA; **(D)** NLRP3 protein; **(E)** Pro Caspase-1 protein; **(F)** Cleaved Caspase-1 protein; **(G)** GSDMD-N protein; **(H)** Representative immunoblot bands.

**FIGURE 5:**
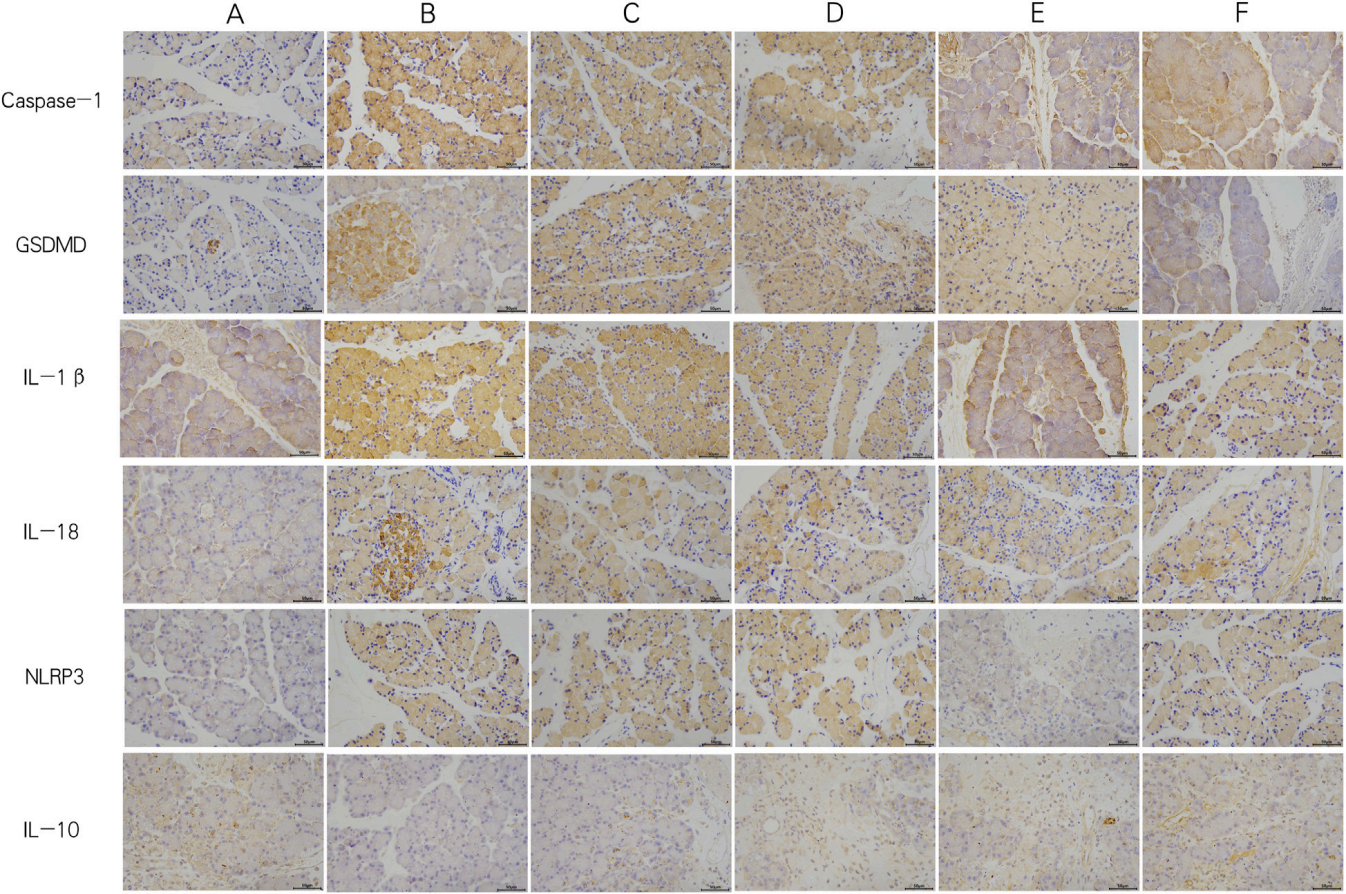
Immunohistochemistry of each group (×400). **(A)** Control; **(B)** PEP model; **(C)** PEP+Indomethacin; **(D)** PEP+GQD; **(E)** PEP+MCC950; **(F)** PEP+MCC950+GQD.

In the PEP model group, the mRNA and protein expression levels of NLRP3, Caspase-1, and GSDMD were significantly increased compared to the control group. The GQD treatment group showed a significant reduction in the expression of NLRP3/Caspase-1 mediated pyroptosis compared to the PEP model group (p < 0.05). There were no significant differences in the mRNA and protein levels of NLRP3, Caspase-1, and GSDMD, as well as pro-Caspase-1 protein, between the GQD and PEP + Indomethacin groups (p > 0.05). Similarly, there were no significant differences in the mRNA levels of Caspase-1 and GSDMD, and the protein levels of NLRP3 and pro-Caspase-1 between the GQD and PEP + MCC950 groups (p > 0.05), as shown in Figure 4. The immunohistochemical showed that the expression of NLRP3/Caspase1 cell apoptosis in the PEP model group was significantly higher than that in other groups, while IL-10 was lower (Figure 5).

### 3.5 Effect of GQD on acinar cells and gene repression validation

Through LDH release rate detection, 400 μM concentration of STC was selected to make cell model. Through CCK8 experiment, 0.5 mg/mL GQD solution was selected as the cell intervention concentration (specific results in Supplementary Table S2a,b). According to qPCR, si-NLRP3-1 silenced NLRP3 with the highest efficiency Of which si-NLRP3-1 sense was GGA​GGA​AGA​AGA​AGA​GAA​ATT, si-NLRP3-1 anti-sense was UUU​CUC​UUC​UUC​UUC​CUC​CTT, si-NC sense was UUC​UCC​GAA​CGU​GUC​ACG​UTT, si-NC anti-sense was ACG​UGA​CAC​GUU​CGG​AGA​ATT.

**FIGURE 6:**
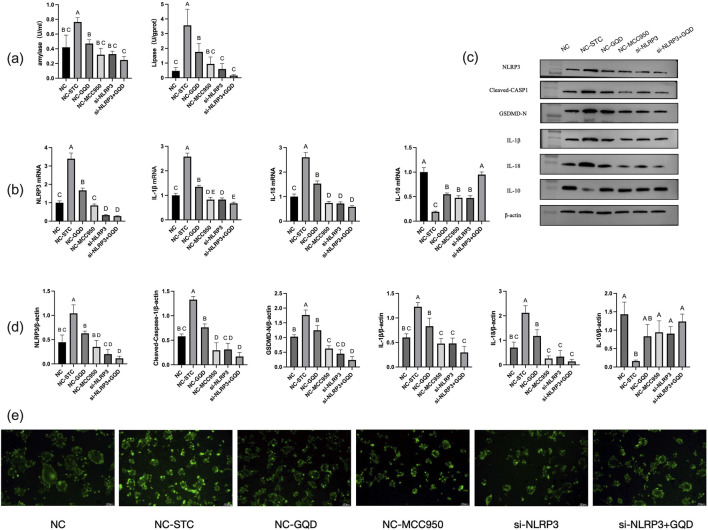
**(a)** Amylase and lipase content; **(b)** PCR analysis; **(c)** Western blot band; **(d)** Western blot analysis; **(e)** Trypsin activity (fluorescent substrate method).

The results of GQD group, negative control (NC) group, STC model group and mcc950 positive control group were similar to those of *in vivo* experiments. In the cell experiment, siRNA group and siRNA+GQD group were added to verify the role of genes. The results showed that both groups significantly improved acinar cell inflammation compared with the model group (Figure 6).

## 4 Discussion

In this study, we validated the preventive effect of GQD in the PEP model, and prophylactic administration of GQD before establishing the PEP model can effectively alleviate subsequent pancreatitis. During this process, the activation of NLRP3 inflammasome and pyroptosis of pancreatic acinar cells were inhibited.

The pathogenesis of PEP is not yet fully understood. It is primarily associated with mechanical injury caused by surgical instruments, chemical injury due to the injection of contrast agents, thermal injury from sphincter of Oddi incision, impaired pancreatic fluid drainage due to papillary edema, and microbial injury resulting from intestinal bacterial translocation (Tryliskyy and Bryce, 2018).

These factors ultimately lead to the activation of pancreatic enzymes, which in turn cause damage to pancreatic acinar cells (PACs) and trigger an inflammatory response. Iohexol, a commonly used contrast agent in clinical ERCP, was injected into the bile-pancreatic duct of rats to simulate the pathogenic process of PEP. The results of this experiment demonstrated that the PEP model group exhibited typical manifestations of acute pancreatitis.

The excessive release of inflammatory cytokines leads to an inflammatory response and oxidative stress, playing a crucial pathological role in the onset and progression of PEP. Inflammatory bodies play a crucial role in promoting and triggering the development of inflammation, autoimmune, and metabolic diseases. NLRP3 inflammasome has become a key mediator of inflammation in many pathologies, leading to the release of pro-inflammatory cytokines IL-1 β and IL-18, as well as GSDMD mediated pyroptosis (Coll et al., 2022). Pancreatitis mainly begins with aseptic local inflammation, triggering systemic inflammatory response syndrome, followed by compensatory anti-inflammatory response syndrome (CARS). Research has found that MCC950 significantly reduces neutrophil infiltration, T cell activation, and disease severity in AP mice (Sendler et al., 2020). NLRP3 inflammasome-dependent pyroptosis is a key factor in the pathogenesis of acute pancreatitis (Al Mamun et al., 2022). In addition, IL-10 can promote mitochondrial phagocytosis, eliminate dysfunctional mitochondria, and lead to dysregulation of NLRP3 inflammasome activation and IL-1β production in the absence of IL-10 signaling transduction.

Currently, multiple studies have been conducted on improving AP through the NLRP3 pathway. A study suggests that high-density lipoprotein (HDL) plays a protective role against acute pancreatitis by inhibiting NLRP3 inflammasome signaling and acinar cell pyroptosis (Lu et al., 2023). Due to the characteristics of multi-target effect and low cost of traditional Chinese medicine, the role of traditional Chinese medicine in anti-inflammatory, antioxidant, immune regulation and other aspects has been paid more and more attention. Many studies have found that some traditional Chinese medicine and its main components have anti-inflammatory, anti-virus, anti-tumor, antioxidant, and antibacterial effects, and can improve respiratory tract infections, digestive system inflammation, and allergic diseases (Liao et al., 2021; Yuan et al., 2022). Iridin (a natural isoflavone) can prevent lipopolysaccharide induced macrophage inflammatory response by inhibiting the glycolytic pathway (Ying et al., 2021), and baicalein can alleviate vascular endothelial cell damage and reduce the production of proinflammatory factors (Ge et al., 2017). Some studies have reported that the widely used formulas (such as Dachengqi Decoction, Qingyi Decoction), single herbs (such as rhubarb, Salvia miltiorrhiza) and monomers (such as baicalein, scutellarin) that are used to treat AP, can all achieve improvements through anti-inflammatory or pancreatic enzyme inhibition effects (Li et al., 2017). Recent studies have found that quercetin can simultaneously solve oxidative stress, inflammation and immune regulation, and has great therapeutic potential in AP (Jiang et al., 2025). A study found that kinsenoside, the main component of Anoectochilus roxburghii (wall.) lindl. (AR), inhibits M1 macrophage polarization through TLR4/STAT1 signaling pathway, thereby alleviating AP (Wang et al., 2025). Yu An et al. found that Qingyi decoction (QYD) can reduce AP by inhibiting NLRP3 inflammasomes. Wogonoside, one of the main chemical components of QYD, can regulate NLRP3 inflammasome activation to prevent AP (An et al., 2025). Baicalin alleviates pyroptosis and inflammatory response in hyperlipidemic pancreatitis by inhibiting the NLRP3/Caspase-1 pathway (Wang et al., 2021).

PEP, as an AP, is characterized by postoperative complications caused by surgery, so preventing the occurrence of diseases is the most important means. Combining the development pattern of diseases to prevent them before they occur is an important treatment concept in traditional Chinese medicine. Guided by this concept, we use GQD to prevent PEP. GQD, as a classic traditional Chinese medicine formula, has been clinically validated for thousands of years for its safety. In this study, the incidence rate of pancreatitis in rats using GQD was reduced and no other obvious complications were found, which to some extent verified the safety of GQD in the prevention of PEP.

This study shows that in PEP rats, the transcription levels of NLRP3, Caspase-1, and GSDMD, as well as the protein expression of NLRP3 and Caspase-1, were significantly elevated, mirroring the changes in amylase, IL-1β, and IL-18. Administration of the NLRP3 inflammasome inhibitor MCC950 markedly reduced pancreatic inflammation in PEP rats, indicating that NLRP3 inflammasome-dependent pyroptosis is also a key factor in the pathogenesis of PEP. Following treatment with GQD, there was a reduction in pancreatic inflammation and the expression of NLRP3, Caspase-1, and GSDMD, similar to the clinical effects observed with indomethacin suppositories and MCC950. Therefore, it is speculated that GQD can effectively prevent PEP, and its primary mechanism may involve the regulation of NLRP3 inflammasome-mediated pyroptosis. Meanwhile, the expression levels of IL-10 were similar among the GQD group, indomethacin group, MCC950 group, and MCC950 plus GQD group, and were significantly higher than those in the PEP model group. To verify this mechanism, siRNA silencing NLRP3 expression was added to the pancreatic acinar cell model with STC intervention, and cellular inflammation was significantly reduced at this time.

Therefore, after GQD intervention, the production of NLRP3 can be inhibited, thereby reducing the synthesis of Caspase-1 and GSDMD, lowering the release of inflammatory factors such as IL-1β and IL-18, and increasing IL-10 levels, thereby preventing the progression of post ERCP pancreatitis.

Although the precise mechanisms by which GQD prevents PEP remain to be further elucidated, multiple components may contribute to its preventive effects. Based on our analysis of GQD components, ammonium glycyrrhizinate, baicalin, berberine, coptisine, daidzein, daidzin, liquiritin, palmatine, wogonin, and wogonoside may play key roles. Among these, previous literature has reported that baicalin can alleviate acute pancreatitis in mice by inhibiting necroptosis through the reduction of phosphorylated MLKL oligomerization (Huang et al., 2022). Daidzein has been shown to alleviate pancreatitis by inhibiting pancreatic enzymes (Zeng et al., 2016). Wogonoside can regulate NLRP3 inflammasome activation to prevent AP (An et al., 2025). Further research is needed to elucidate the roles of these components in the prevention of PEP, including changes in their absorption into the bloodstream and validation of their therapeutic efficacy *in vitro* cells. Although the role of GQD in preventing PEP has been explored in rat models and *in vitro* models, there is still a lack of direct evidence for its role in human PEP prevention. Although the rat model is a classic model for the study of acute pancreatitis, and our modeling method simulates the stimulation of ERCP surgery by injecting contrast agent into the rat pancreaticobiliary duct, it is still different from clinical ERCP surgery, and may not fully simulate the complex pathological environment of human PEP. *In vitro* experiments using pancreatic acinar cells can analyze specific signaling pathways, but can not reflect the overall mechanism of multi organ interaction, neuroendocrine regulation, etc., while the multi-target characteristics of traditional Chinese medicine may not be fully reflected in such simplified models. Next, randomized controlled clinical studies with large samples are needed to comprehensively evaluate the role of GQD in preventing pep. In clinical applications, the effects of different doses of GQD and the expression of NLRP3 inflammasome in patients also need further exploration.

## 5 Conclusion

In summary, GQD can effectively prevent PEP, and its primary mechanism may be related to the regulation of NLRP3 inflammasome-mediated pyroptosis.

## Data Availability

The original contributions presented in the study are included in the article/Supplementary Material, further inquiries can be directed to the corresponding author.
