# Supplementary material for: Gegen Qinlian decoction prevents post-ERCP pancreatitis by regulating NLRP3 inflammasome-mediated pyroptosis
Source: Front Pharmacol. 2025 Jun 20;16:1588585. doi: 10.3389/fphar.2025.1588585 (PMC12226573; doi:10.3389/fphar.2025.1588585)
Supplement: Supplementary file 1 [file Supplementaryfile1.docx]

## Histopathological Scoring

|  | (A) Control | | (B)PEP model | | | (C) PEP+Indomethacin | | (D) PEP+GQD | | | | (E) PEP+MCC950 | | (F) PEP+MCC950+GQD | | |
| --- | --- | --- | --- | --- | --- | --- | --- | --- | --- | --- | --- | --- | --- | --- | --- | --- |
| Edema | 0 | 0 | 1 | 1 | 2 | 0.5 | 2 | 0.5 | 0 | 0 | 2.5 | 0 | 0 | 0 | 0 | 0 |
| Acinar necrosis | 0 | 0 | 0 | 0 | 4 | 0.5 | 0.5 | 4 | 0 | 0 | 0 | 0 | 0 | 0 | 0 | 4 |
| Hemorrhage and fat necrotis | 0 | 1 | 3.5 | 1.5 | 0.5 | 0 | 0.5 | 0 | 0.5 | 1 | 0.5 | 0.5 | 0.5 | 0 | 0.5 | 0 |
| Inflammation and perivascular infltrate | 0 | 0 | 4 | 4 | 4 | 2 | 1.5 | 0 | 0 | 4 | 0.5 | 0 | 1 | 0 | 0 | 0.5 |
| **Total** | 0 | 1 | 8.5 | 6.5 | 10.5 | 3 | 4.5 | 4.5 | 0.5 | 5 | 3.5 | 0.5 | 1.5 | 0 | 0.5 | 4.5 |
