# Supplementary material for: Gegen Qinlian decoction prevents post-ERCP pancreatitis by regulating NLRP3 inflammasome-mediated pyroptosis
Source: Front Pharmacol. 2025 Jun 20;16:1588585. doi: 10.3389/fphar.2025.1588585 (PMC12226573; doi:10.3389/fphar.2025.1588585)
Supplement: Supplementary file 2 [file Supplementaryfil4.docx]

**Supplementary Table 2a. LDH release rate (%)**

| **Sample control** | **Maximum enzyme activity control** | **100μM STC** | **200μM STC** | **400μM STC** | **800μM STC** | **1600μM STC** |
| --- | --- | --- | --- | --- | --- | --- |
| -0.007860095 | 1.026349423 | 0.161675116 | 0.294465982 | 0.441123844 | 0.405210242 | 0.440868232 |
| -0.023005155 | 0.99561198 | 0.115217484 | 0.296191369 | 0.377412346 | 0.450517616 | 0.486750735 |
| 0.030865249 | 0.978038598 | 0.105120777 | 0.28027947 | 0.365654156 | 0.42987688 | 0.498764538 |

**Supplementary Table 2b. Cell viability (%)**

| **10%FBS** | **control** | **0.1mg/mL GQD** | **0.2mg/mL GQD** | **0.5mg/mL GQD** | **1mg/mL GQD** | **2mg/mL GQD** |
| --- | --- | --- | --- | --- | --- | --- |
| 101.3347291 | 51.24313007 | 58.3093431 | 66.16069092 | 79.82203612 | 72.75582308 | 85.78906046 |
| 105.260403 | 53.59853442 | 59.72258571 | 63.96231353 | 79.5079822 | 82.0204135 | 86.26014132 |
| 101.3347291 | 56.26799267 | 57.83826224 | 64.59042136 | 80.76419785 | 82.49149437 | 86.73122219 |
| 86.73122219 | 55.48285789 | 60.19366658 | 66.47474483 | 80.13609003 | 74.4831196 | 88.61554567 |
| 102.9049987 | 45.11907878 | 58.78042397 | 66.63177179 | 81.39230568 | 80.6071709 | 89.71473436 |
| 102.4339178 | 56.8961005 | 59.25150484 | 67.57393353 | 79.19392829 | 82.49149437 | 89.24365349 |
