# Supplementary material for: Gegen Qinlian decoction prevents post-ERCP pancreatitis by regulating NLRP3 inflammasome-mediated pyroptosis
Source: Front Pharmacol. 2025 Jun 20;16:1588585. doi: 10.3389/fphar.2025.1588585 (PMC12226573; doi:10.3389/fphar.2025.1588585)

Berberine hydrochloride


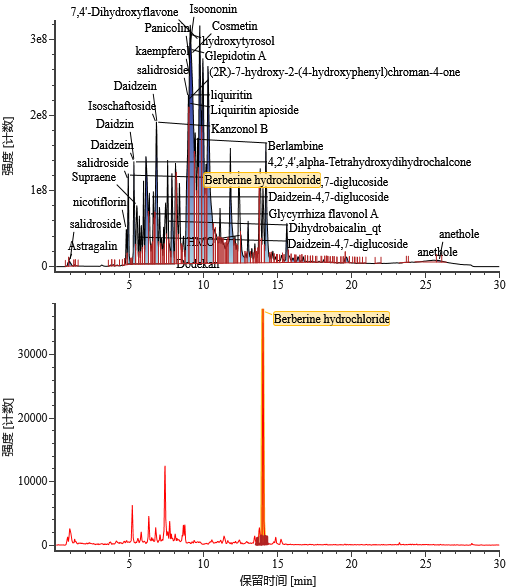


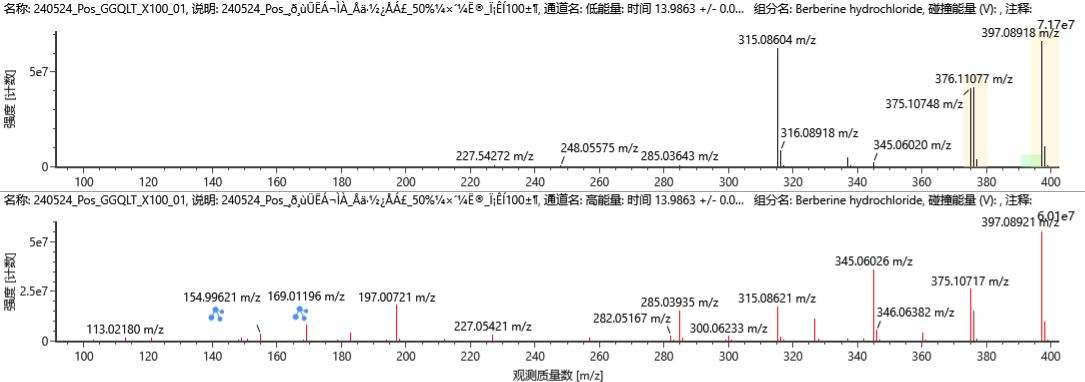


Baicalin


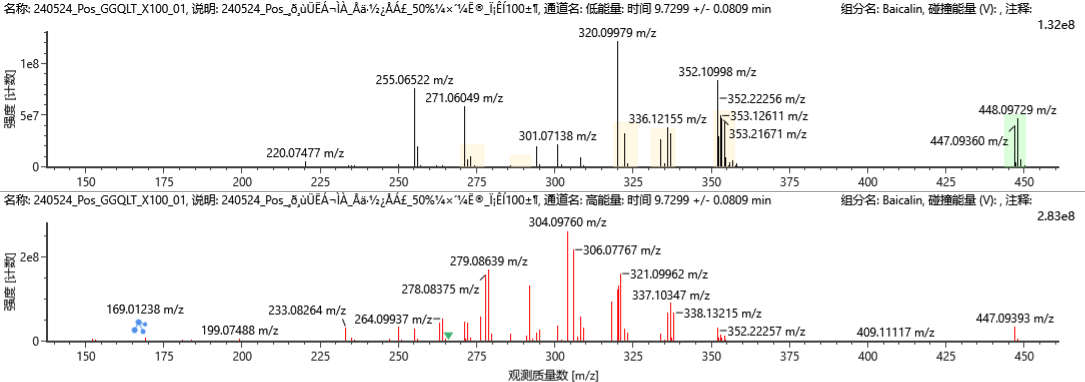


coptisine


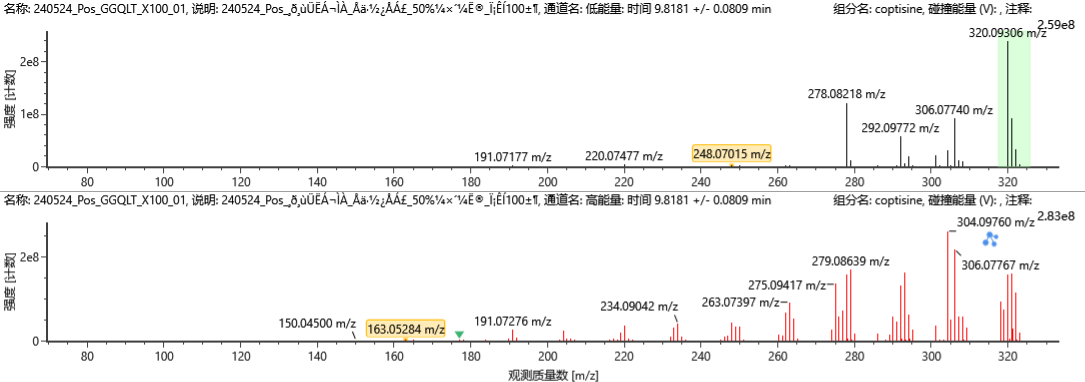


Wogonin


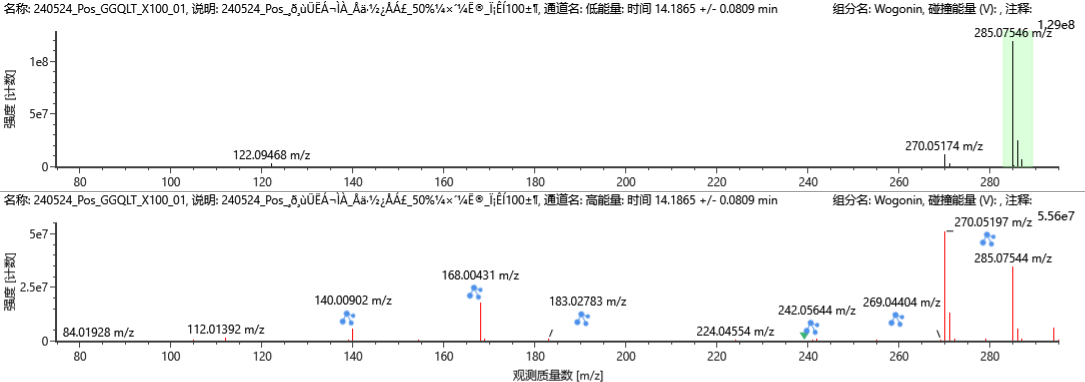


wogonoside


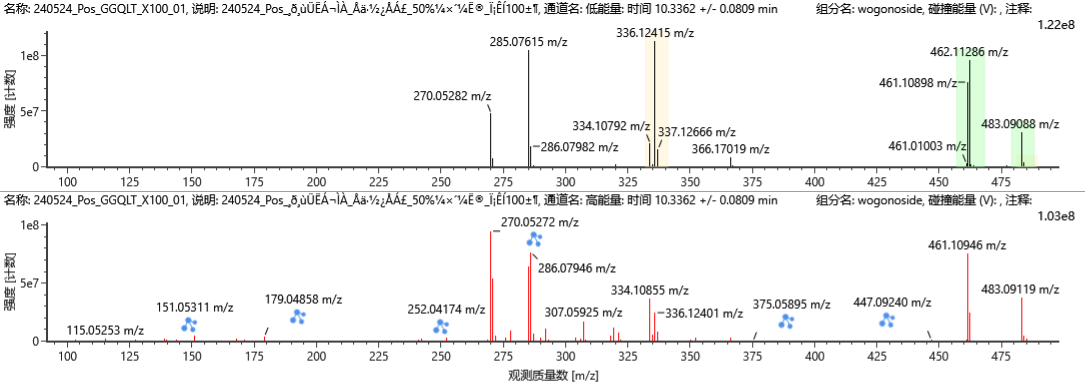


Ammonium glycyrrhizinate


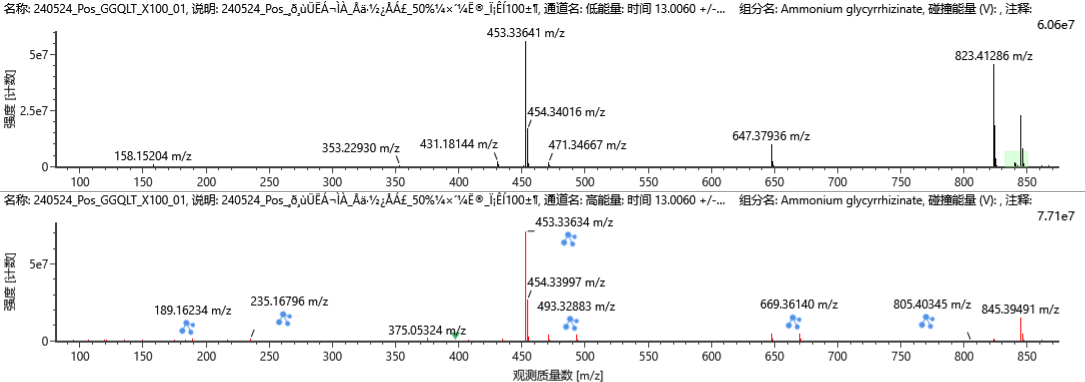


liquiritin


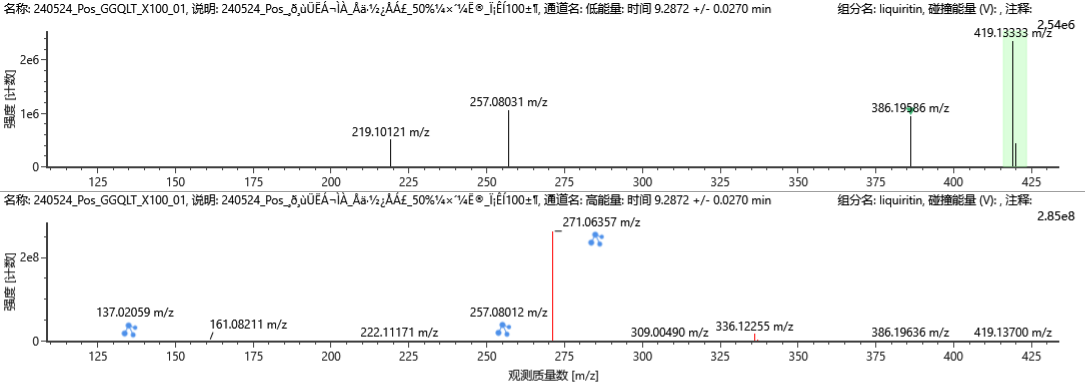


Daidzein


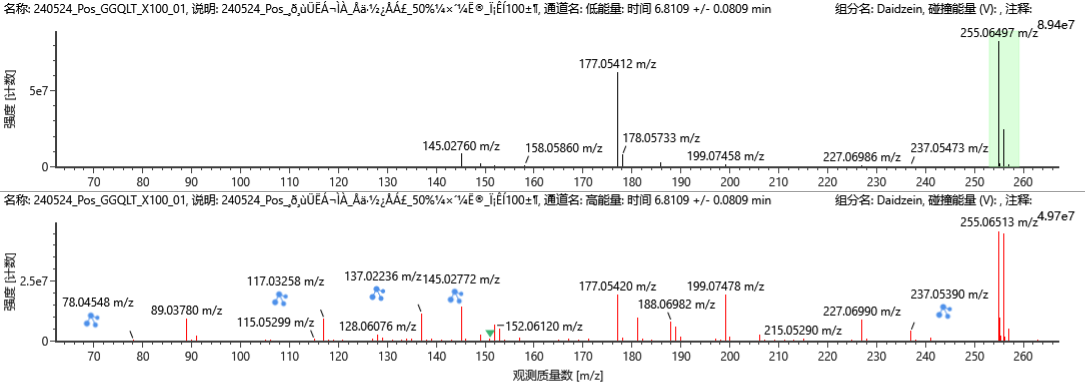


Daidzin


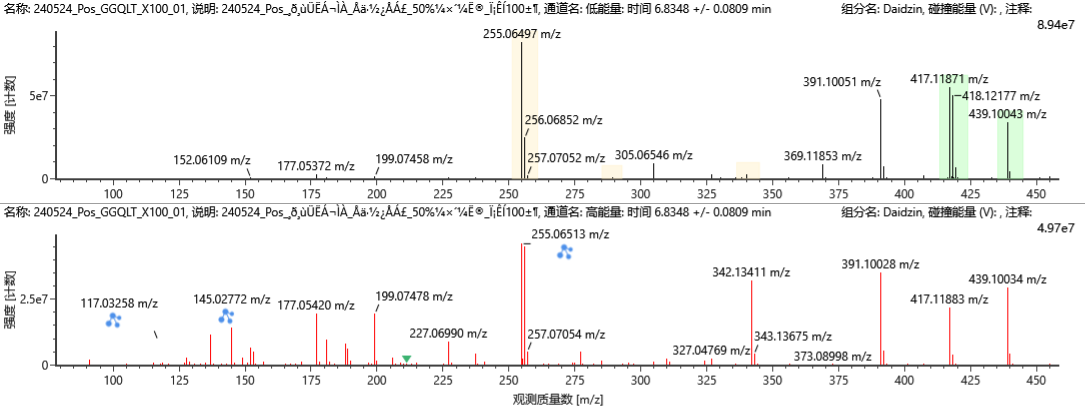


Palmatine


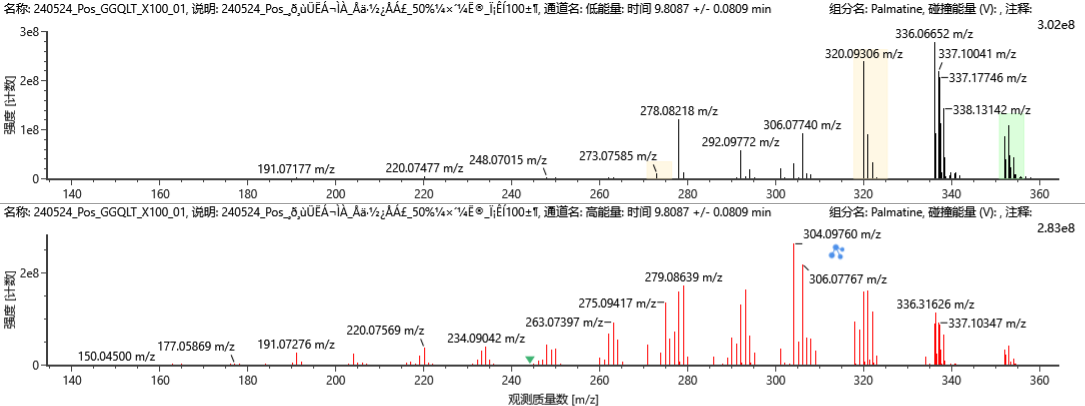


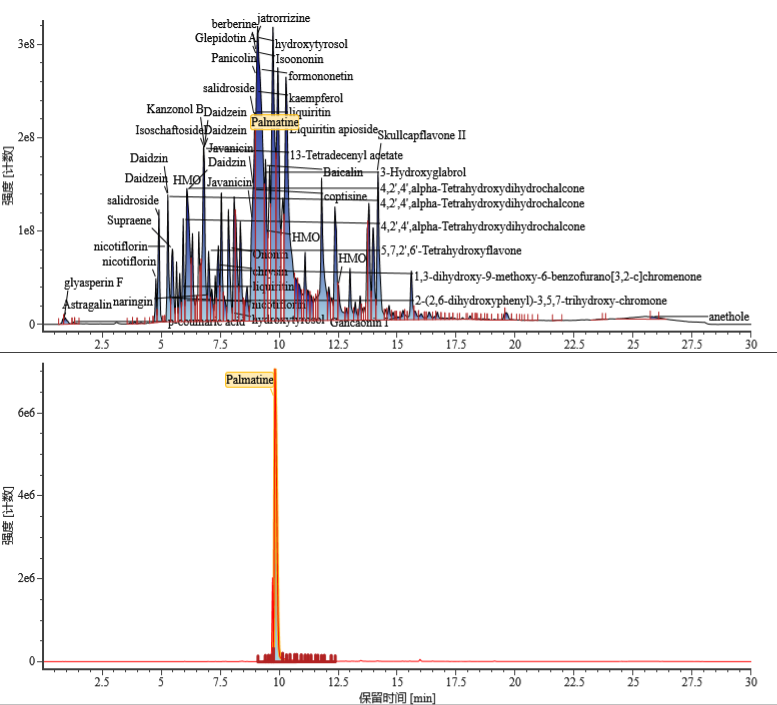

Supplement: Supplementary file 3 [file Supplementaryfile2.docx]
